# Comparison of tourniquet application only during cementation and long-duration tourniquet application in total knee arthroplasty: a meta-analysis

**DOI:** 10.1186/s13018-018-0927-6

**Published:** 2018-08-30

**Authors:** Cong Wang, Chenhe Zhou, Hao Qu, Shigui Yan, Zhijun Pan

**Affiliations:** Department of Orthopaedic Surgery, The Second Affiliated Hospital, Zhejiang University School of Medicine, No. 88 Jiefang Road, Hangzhou, 310009 China

**Keywords:** Tourniquet, Total knee arthroplasty (TKA), Cementation, Meta-analysis

## Abstract

**Background:**

Tourniquet is widely used by orthopedic surgeons in total knee arthroplasty (TKA). However, there are still controversies on the optimal timing of tourniquet application. The aim of this meta-analysis was to compare the effect and safety of tourniquet application only during cementation with long-duration tourniquet application in TKA.

**Methods:**

An electronic literature search of PubMed, the Cochrane library, Embase, and Web of Science was conducted in July 2017. All randomized controlled trials (RCTs) comparing tourniquet application only during cementation with long-duration tourniquet application in TKA were included. RevMan 5.3 software was selected to perform the meta-analysis.

**Results:**

Seven studies involving 440 TKAs were included for meta-analysis. The results suggested that although significant less intraoperative and total blood loss were observed with long-duration tourniquet application, tourniquet application only during cementation would not increase the number of transfusion and operation time. Tourniquet application only during cementation results in less knee pain on post-operative day 1 (POD 1), less time needed to achieve straight-leg raise, and less minor complications following TKA.

**Conclusions:**

Tourniquet application only during cementation might reduce the rate of minor complications and have faster functional recovery during the early rehabilitation period following TKA, but it could not limit intraoperative and total blood loss. No definitive conclusions can be drawn based on the current evidences. Further, large well-designed RCTs with extensive follow-up are still needed to validate this research.

## Background

Total knee arthroplasty (TKA) is a successful procedure for reducing pain and restoring function in patients with end-stage osteoarthritis. TKA has been reported to be associated with a significant amount of blood loss, sometimes necessitating blood transfusion [1]. Although the application of tourniquet is still highly controversial [2], considering the fact of providing clearer surgical visualization, less intraoperative blood loss, thus ensuring better cementation, it has become a common practice and is widely used by orthopedic surgeons during TKA.

However, tourniquet’s use may also be associated with several complications, including thigh pain, limb swelling, nerve palsy, vascular injuries, subcutaneous thigh fat necrosis, postoperative stiffness, delayed recovery of quadriceps strength, wound complications, and deep vein thrombosis (DVT) [3–8].

There are still controversies on the optimal timing of tourniquet application, which may have vital influence on clinical outcomes following TKA. It is widely accepted that the prolonged duration of tourniquet application might be a crucial factor for complications [6, 8, 9], which suggests longer ischemic time for tissues. Hence, it is important to minimize the tourniquet time. Some investigators begin to focus on whether the limited application of tourniquet (tourniquet application only during the cementation) in TKA could reduce the complications and facilitate functional recovery. Fan et al. [10] demonstrated that limited use of a tourniquet in TKA provides the benefit of decreased limb swelling and knee joint pain while not compromising the operation time or blood loss and recovery. Meanwhile, Wang et al. [11] showed tourniquet application only during cementation would reduce postoperative and hidden blood losses without increasing the allogeneic blood transfusion rate. In addition, short-duration tourniquet use would result in faster recovery and less pain during the early rehabilitation period following TKA. In contrast, Mittal et al. [12] reported restricting tourniquet application to the period of cementing is associated with a significantly higher risk of transfusion, and indicated the approach is impractical if it is not offset by gains in functional recovery. Thus, this has to be balanced against the increased blood loss and risk of transfusion when using the tourniquet only during the cementation.

Therefore, whether tourniquet application only during cementation in TKA is beneficial remains debatable. And there has been no meta-analysis evaluating tourniquet application only during cementation compared with the long-duration tourniquet application in TKA. Accordingly, we systematically reviewed the current randomized controlled trials (RCTs) to investigate which tourniquet application strategy is better. Our hypothesis is that tourniquet application only during cementation may result in less knee pain, faster functional recovery during the early rehabilitation, and less complications, but may increase intraoperative and total blood loss.

## Methods

### Search strategy

This meta-analysis was preformed according to the Preferred Reporting Items for Systematic Reviews and Meta-Analyses guidelines (the PRISMA statement) [13]. The electronic databases of PubMed, the Cochrane library, Embase, and Web of Science were systematically searched for relevant academic clinical trials comparing tourniquet application only during cementation to long-duration tourniquet application in TKA from inception to July 2017. The following search terms were used to maximize scope of the search: tourniquet and (total knee arthroplasty or total knee replacement). Furthermore, the reference lists of the identified articles were reviewed to search for additional studies of interest that potentially met the study criteria, and no restriction was made on the language of the publication.

### Inclusion and exclusion criteria

We identified literature that met the following inclusion criteria: (1) patients underwent primary TKA, (2) randomized controlled trials, (3) comparison of tourniquet application only during cementation and long-duration tourniquet application in total knee arthroplasty. The tourniquet inflated before skin incision and deflated following the completion of cementation or the wound closure was considered as long-duration tourniquet application. (4) Outcome measurements should include at least one of these parameters (tourniquet time, surgery time, calculated blood loss, total measured blood loss, intraoperative blood loss, postoperative blood loss, number of transfusion, knee pain, time needed to achieve straight-leg raise and complications).

Exclusion criteria were (1) non-randomized controlled trials, (2) unpublished data, (3) proceedings of meetings, (4) revision TKA, (5) different tourniquet application strategy.

### Data extraction

Two researchers independently extracted the data from the individual study using the same format, after which the data were checked by a third author and any disagreement was resolved by consensus. Whenever necessary, we contacted the authors of the studies for the missing data and additional information. Relevant data extracted included publication information; demographic characteristic; tourniquet time; operative time; blood loss measures, including the intraoperative blood loss, postoperative blood loss, total measured blood loss, and calculated blood loss; number of transfusion; time needed to achieve straight-leg raise; knee pain scores; complications (including minor complications and major complications). Total measured blood loss was defined as the sum of intraoperative and measured postoperative blood loss, the calculated blood loss was estimated with formulas [14]. The complication was distinguished as a minor or major one according to whether a second operation was needed. We defined minor complications as wound complications such as oozing, erythema, marginal necrosis, superficial infection, slight knee stiffness, significant leg swelling, and DVT, which could be healed through conservative treatment and did not need another operation. We defined major complications as vessel injuries, infections, wound dehiscence, active hemorrhage and hematomas that required drainage or debridement or revision, and serious knee stiffness which need manipulation with the patient under anesthesia.

### Quality assessment

Two authors independently assessed the risk of bias of included studies, with the following items: randomization, allocation concealment, blinding of participants, blinding of outcome assessment, incomplete outcome data, selective outcome reporting, and other bias [15]. Based on the information provided from included studies, each item was recorded by “low,” “high,” or “unclear”. Low indicates low risk of bias, high indicates high risk of bias, unclear indicates lack of information or unknown risk of bias.

### Statistical analysis

The software of RevMan 5.3 was used to perform meta-analysis. Odds ratios (OR) with 95% confidence interval (95% CI) was calculated for dichotomous outcomes, and mean difference (MD) with 95% CI were used for continuous outcomes. *p* values less than 0.05 were considered statistically significant (*p* < 0.05). Heterogeneity among studies was tested using *I*^2^ statistic and substantial heterogeneity was represented by an *I*^2^ value greater than 50%. If significant heterogeneity was found in the meta-analysis, we used a random effect model; otherwise, we used a fixed effect model.

## Results

### Study selection

**Fig. 1:**
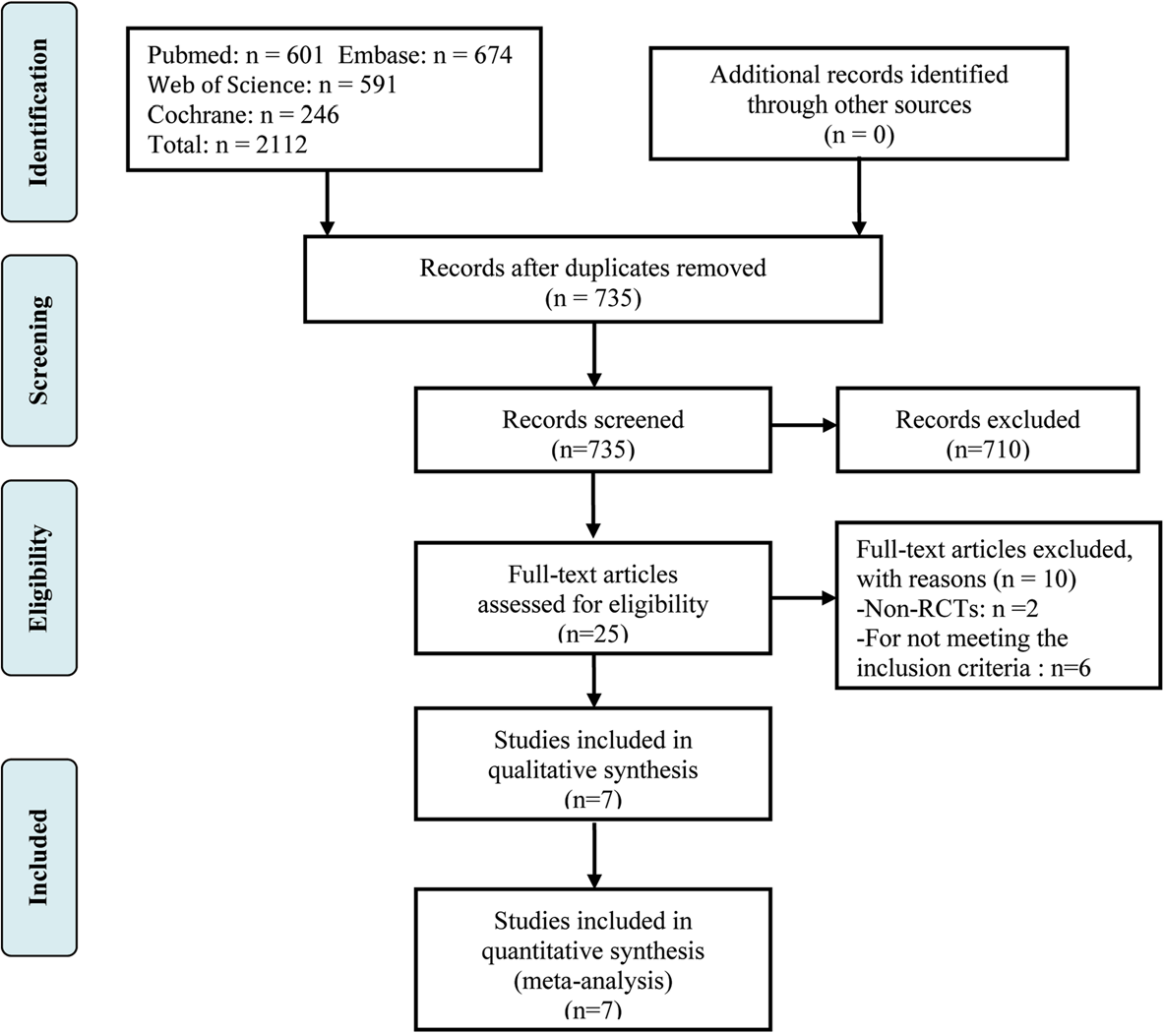
The flow chart of literature screening

A flow chart of literature screening is shown in Fig. 1. From the initial database search, a total of 2250 relevant trials were yielded. After removing 1402 duplicates, 848 studies were reserved for title or abstract reviewing according to the inclusion criteria. The remaining 25 articles were subjected to full-text screen. Finally, seven studies were considered to be eligible for meta-analysis [10–12, 16–19].

### Study characteristics

The characteristics of the included studies are showed in Table 1. The studies were published between 1997 and 2016. A total of 440 TKAs were performed in 377 adult patients. The mean age ranged from 63.27–72.5, and the mean body mass index (BMI) ranged from 26.26–32.6. The patients’ parameters (age, BMI, preoperative knee function) were reported similar between groups. The results of the quality assessment are summarized in Table 2. Regarding the risk of bias, the overall quality of the included studies was considered adequate.

**Table 1:** Characteristics of included studies

Author/year	Patients	Knees		Gender (male/female)		Mean age (years)		BMI		Operative time (min)		Tourniquet time (min)	
		Cementation	Long	Cementation	Long	Cementation	Long	Cementation	Long	Cementation	Long	Cementation	Long
Fan 2014	60	30	30	9/21	7/23	63.27 ± 7.39	65.37 ± 7.11	26.26 ± 1.52	27.24 ± 2.69	111.25 ± 20.04	120.81 ± 8.12	23.20 ± 5.30	75.03 ± 133.99
Hakkalamani 2015	55	30	30	13/17	17/13	66.7 ± 8.5	69 ± 8.5	31.9 ± 6.2	31.5 ± 5.5	73.5 ± 13	76.7 ± 19	14 ± 4	76.7 ± 19
Harvey 1997	52	16	36	NA	NA	72.4	68.3	NA	NA	NA	NA	NA	NA
Kvederas 2013	24	12	12	3/9	1/11	67.3 ± 6.4	72.9 ± 4.5	31.1 ± 4.8	31.8 ± 4.1	59.8 ± 5.0	60.6 ± 8.7	11	60
Mittal 2012	65	31	34	6/25	9/25	67.5 ± 8.9	66.6 ± 8.4	32.5 ± 5.6	32.6 ± 5.6	105 ± 18.4	103 ± 16.6	22.5 ± 14.4	76.4 ± 15.1
Tarwala 2014	71	40	39	13/22	14/22	64.6 ± 9.3	66.1 ± 9.8	31.4 ± 6.4	29.9 ± 5.3	90 ± 23	86 ± 22	9(7–14)	43(24–62)
Wang 2016	50	25	25	4/21	5/20	72.5 ± 6.8	72.3 ± 7.1	29.1 ± 6.6	28.8 ± 6.2	93.8 ± 22.7	84.7 ± 12.4	10.9 ± 1.8	54.8 ± 6.7

*NA*, not available

**Table 2:** Risk of bias in included studies

Author/year	Randomization	Allocation concealment	Blinding of participants	Blinding of outcome assessment	Incomplete outcome data	Selective outcome reporting	Other bias
Fan 2014	Low	Unclear	Low	Low	Low	Low	Unclear
Hakkalamani 2015	Low	Low	Low	Low	Low	Low	Unclear
Harvey 1997	Unclear	Unclear	Low	Low	Low	Low	Unclear
Kvederas 2013	Low	Low	Low	Low	Low	Low	Unclear
Mittal 2012	Low	Low	Low	Low	Low	Low	Unclear
Tarwala 2014	Low	Unclear	Low	Low	Low	Low	Unclear
Wang 2016	Low	Low	Low	Low	Low	Low	Unclear

### Blood loss

**Fig. 2:**
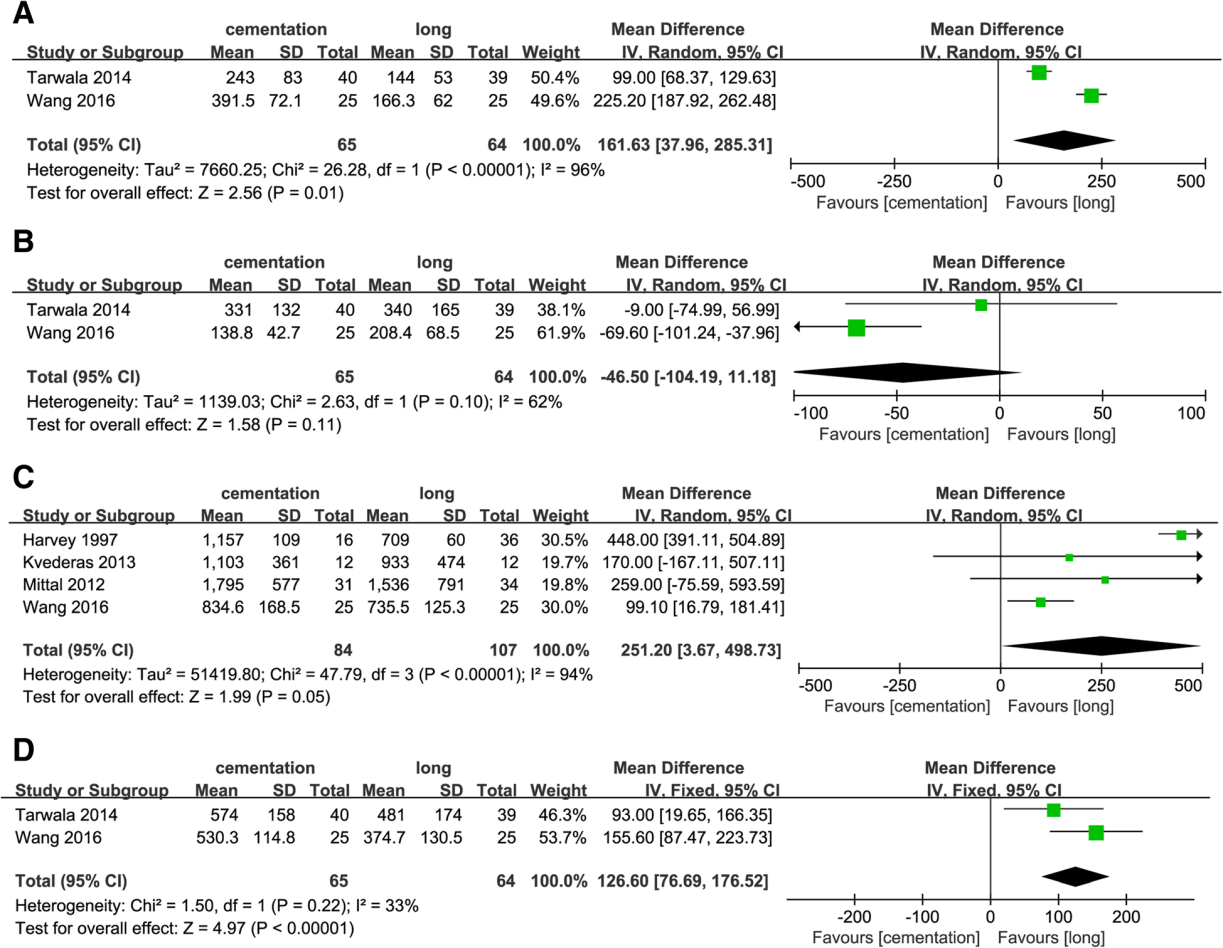
**a** Forest plot of intraoperative blood loss. **b** Forest plot of postoperative blood loss. **c** Forest plot of calculated blood loss. **d** Forest plot of total measured blood loss

Two studies [11, 19] including 129 knees were included for analysis of the intraoperative blood loss and demonstrated significant more blood loss in the group with tourniquet application only during cementation (MD = 161.63; 95% CI 37.96 to 285.31; *p* = 0.01) (Fig. 2a). In two studies [11, 19], postoperative blood loss showed no significant difference between the groups (MD = − 46.50; 95% CI − 104.19 to 11.18; *p* = 0.11) (Fig. 2b). In addition, a total of four studies [11, 12, 17, 18] addressed the calculated blood loss, the pooled results showed tourniquet application only during cementation significantly increased the blood loss compared to that of long-duration tourniquet application (MD = 251.20; 95% CI 3.67 to 498.73; *p* = 0.05) (Fig. 2c). Two studies [11, 19] in which the total measured blood loss was measured also showed significant difference between the two groups (MD = 126.60; 95% CI 76.69 to 176.52; *p* < 0.00001) (Fig. 2d).

### Number of transfusion

**Fig. 3:**
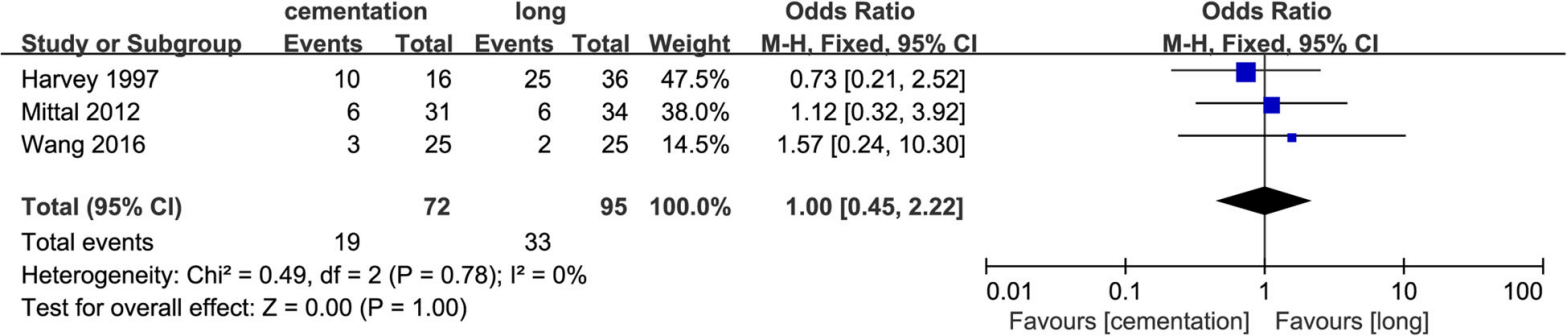
Forest plot of number of transfusion

Three included studies [11, 12, 17] investigated the number of transfusion after TKA. The combined data showed no significant difference between the groups (OR = 1.00; 95% CI 0.45 to 2.22; *p* = 1.00) (Fig. 3).

### Operation time

**Fig. 4:**
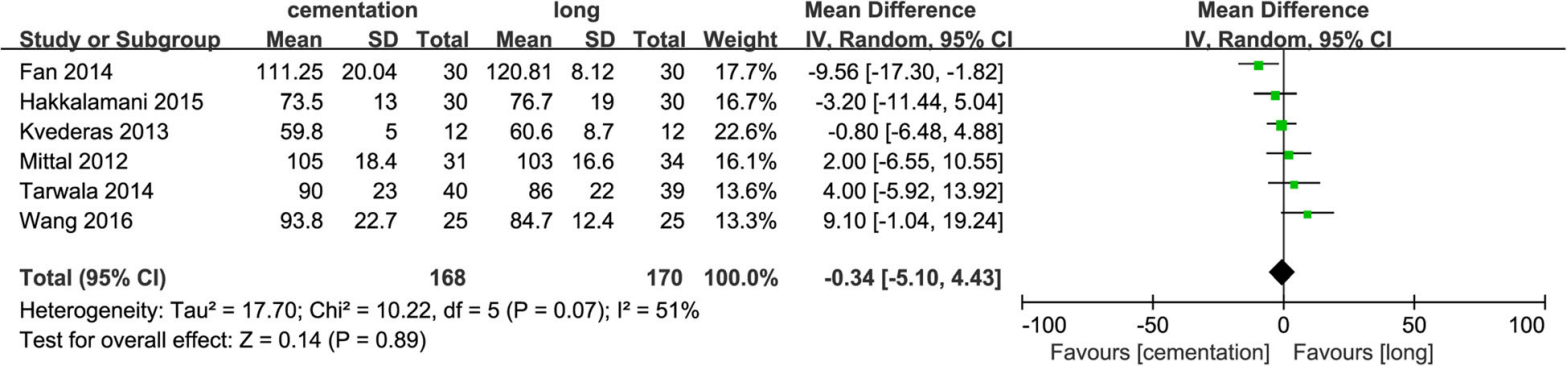
Forest plot of operation time

Six studies [10–12, 16, 18, 19] involving the operation time during TKA showed no difference between the groups (MD = − 0.34; 95% CI − 5.10 to 4.43; *p* = 0.89) (Fig. 4).

### Tourniquet time

**Fig. 5:**
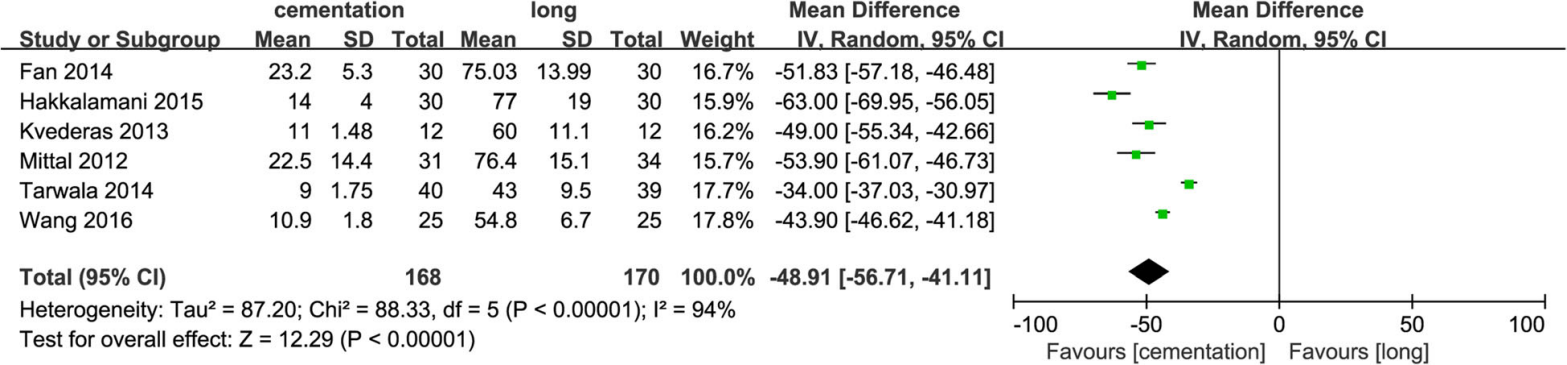
Forest plot of tourniquet time

Six studies [10–12, 16, 18, 19] providing the tourniquet time indicated that it was significantly shorter in the group with tourniquet use only during cementation (MD = − 48.91; 95% CI − 56.71 to − 41.11; *p* < 0.00001) (Fig. 5).

### Knee pain

**Fig. 6:**
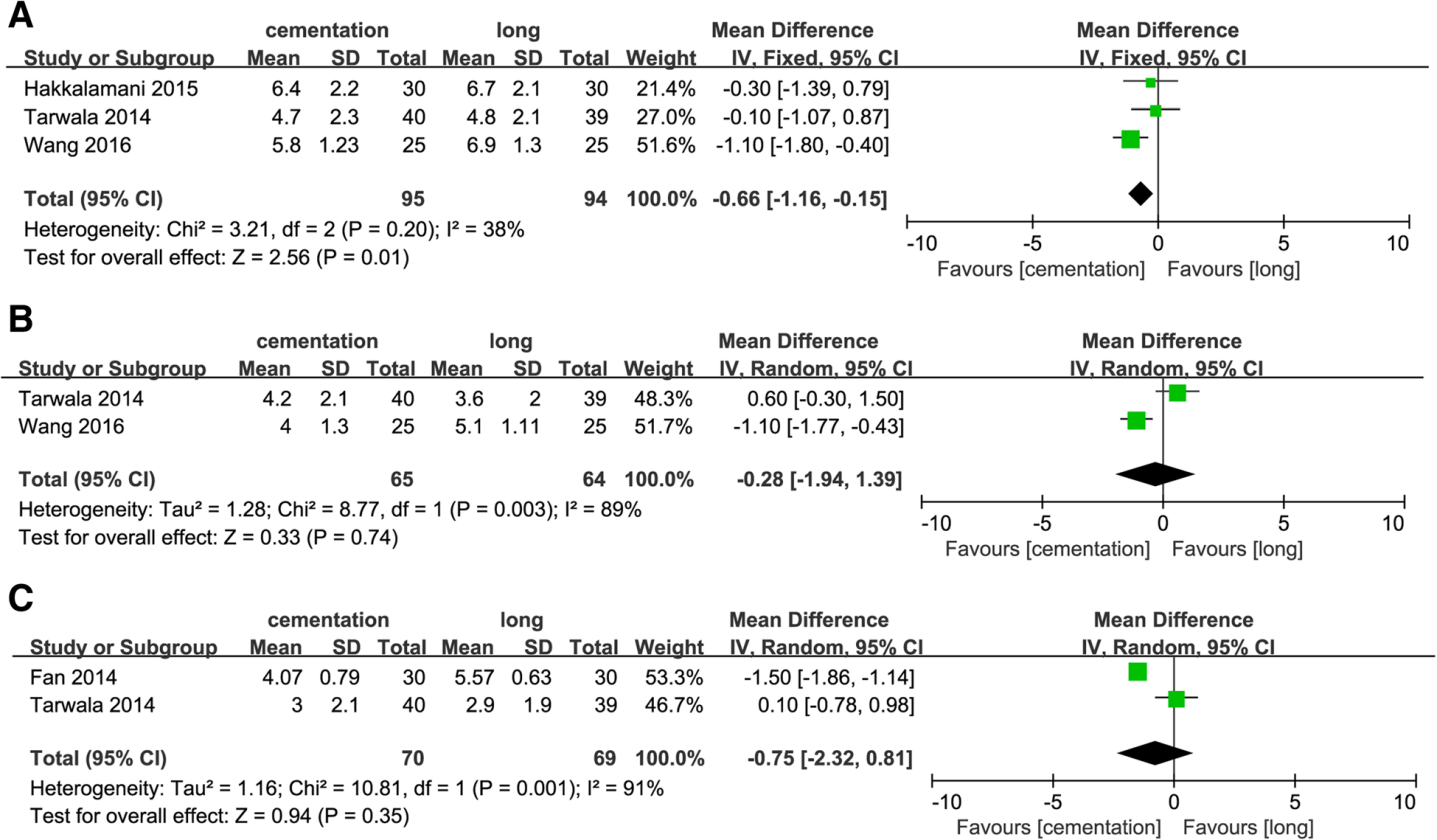
**a** Forest plot of VAS knee pain scores on POD 1. **b** Forest plot of VAS knee pain scores on POD 2. **c** Forest plot of VAS knee pain scores on POD 3

Three included studies [11, 16, 19] investigated visual analogue scale (VAS) knee pain scores on post-operative day 1 (POD 1). The pooled results showed less knee pain scores in the group tourniquet application only during cementation (MD = − 0.66; 95% CI − 1.16 to − 0.15; *p* = 0.01) (Fig. 6a). Of these seven RCTs, two studies [11, 19] reported the VAS knee pain scores on POD 2. Analysis of two studies showed no difference (MD = − 0.28; 95% CI − 1.94 to 1.39; *p* = 0.74) (Fig. 6b). Only two studies [10, 19] investigated VAS knee pain score on POD 3. There was no difference between the groups (MD = − 0.75; 95% CI − 2.32 to 0.81; *p* = 0.35) (Fig. 6c).

### Straight-leg raise

**Fig. 7:**
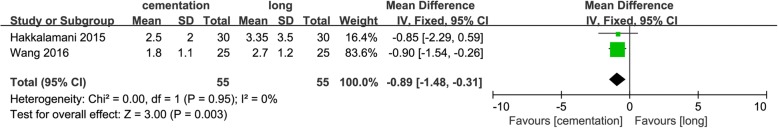
Forest plot of the time needed to achieve straight-leg raise

Two studies [11, 16] assessed the time needed to achieve straight-leg raise after TKA, and the results showed that the group tourniquet application only during cementation was associated with significant less time needed to achieve straight-leg raise compared to long-duration tourniquet application group (MD = − 0.89; 95% CI − 1.48 to − 0.31; *p* = 0.003) (Fig. 7).

### Complications

**Fig. 8:**
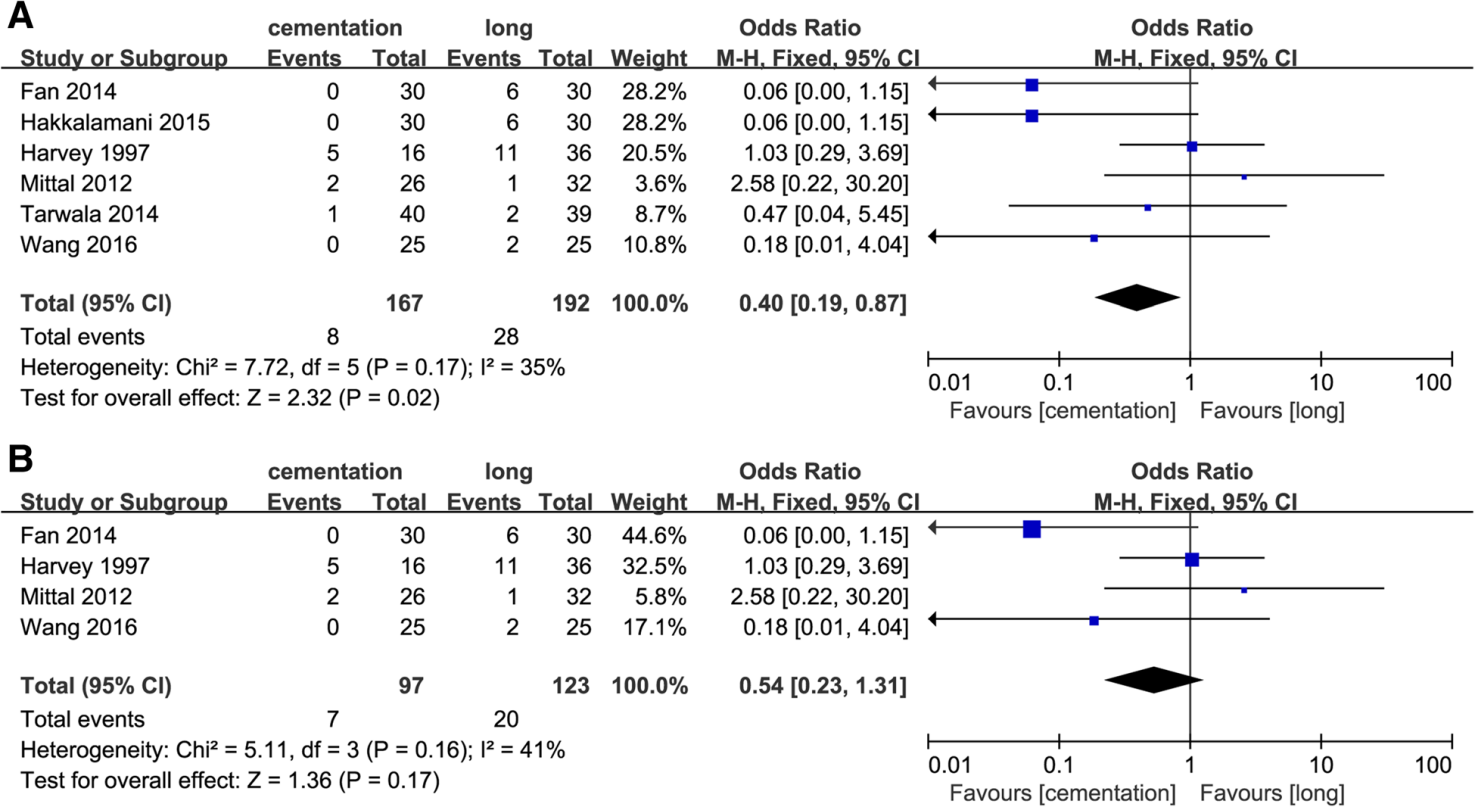
**a** Forest plot of minor complications. **b** Forest plot of deep vein thrombosis (DVT)

Six studies [11, 12, 16, 17, 19] reported complications including minor complications and major complications. From Fig. 8a, we could draw the conclusion that tourniquet application only during cementation significantly decreased the risk of minor complications (OR = 0.40; 95% CI 0.19 to 0.87; *p* = 0.02). With regard to DVT, a subgroup analysis was conducted and the result indicated no significant difference between the two groups (OR = 0.54; 95% CI 0.23 to 1.31; *p* = 0.17) (Fig. 8b). Only one study reported major complications which could not be pooled in meta-analysis [19].``

## Discussion

To our knowledge, this is the first meta-analysis of RCTs comparing the effect and safety of tourniquet application only during cementation with long-duration tourniquet application in TKA. The most important finding of the meta-analysis was that although significant less intraoperative and total blood loss were observed with long-duration tourniquet application, tourniquet application only during cementation would not increase the number of transfusion and operation time. Besides, tourniquet application only during cementation result in less knee pain, less time needed to achieve straight-leg raise, and less minor complications following TKA.

For blood loss, the result demonstrates that long-duration tourniquet application significantly reduced the intraoperative blood loss, calculated blood loss, and total measured blood loss, which were consistent with some previous studies [20–23]. Deflating the tourniquet after the cementation theoretically allows a better control of wound bleeding and patients would have better hemostasis, thus leading to less blood loss. However, rapid reactive hyperemia and increase in fibrinolytic activity have been demonstrated to occur in the first period after tourniquet releasing, leading to excessive bleeding [24–27]. The higher perioperative blood loss caused by fibrinolytic activity can probably be controlled by a closed wound and pressure dressing [28]. Releasing tourniquet after cementation provides a window of time to the activation of fibrinolytic leading to increasing bleeding [29]. Based on this, tourniquet application only during cementation theoretically would result in more perioperative blood loss.

No significant difference was found in the incidence of transfusion between these two groups. However, differences were obviously detected between the groups in perioperative blood loss. Theoretically, the transfusion is supposed to be associated with the loss of blood. Moráis et al. [30] reported perioperative transfusion rate had no significant relevance with application time of tourniquet in TKA but was relevant with preoperative hemoglobin level and body mass index. Moreover, no difference in transfusion rate between the groups also may result from the variability in the criteria for transfusion.

Postoperative functional recovery for TKA is particularly important. The current study showed that tourniquet application only during cementation significantly reduced VAS knee pain and the time needed to achieve straight-leg raise following TKA. Fan et al. [10] also discovered the limited use of a tourniquet in TKA provides the benefit of decreased limb swelling and better active knee flexion while not compromising the operation time or blood loss and recovery. The possible reason is the direct damage of the tourniquet and reperfusion injury might increase pain that would hamper patients’ postoperative rehabilitation [31]. And additional limb swelling in the long-duration tourniquet group after TKA might cause an increased weight in the affected limb sufficient to require more muscle strength for performing straight-leg raise. Furthermore, Dennis et al. [32] indicated that patients who underwent TKA using a tourniquet had diminished quadriceps strength during the first 3 months after TKA. Early mobilization after TKA may be delayed in the patients with quadriceps weakness. It therefore seems to be highly desirable to keep the duration of tourniquet to a minimum [33–36]. The tourniquet application only during cementation may be considered superior since it hardly influences the functional recovery after TKA.

As for complications, this study shows that tourniquet application only during cementation reduced the risks of minor complication. With respect to major complication, only one study reported one patient suffered a compartment syndrome in the long-duration tourniquet application group [19]. Previous studies have showed that the prolonged duration of tourniquet application might be a crucial factor for complications [6, 8, 9, 37]. In our included studies, the most common minor complications were wound complications. It is well known that oxygen supply in the soft tissue around the incision is one of the key elements for sound incision healing. Clarke et al. [38] indicated that tourniquet sharply decreased the oxygen content in the soft tissue around the incision for ischemia-reperfusion injuries in TKA. Longer tourniquet application would further aggravate the soft tissue hypoxia around the incision, cause more excessive inflammation and muscle damage, and consequently increase the risk of wound complications. It was also reported that every additional 10 min of tourniquet time was associated with an increased risk for complications [39]. Therefore, it is important to shorten the duration of tourniquet time to minimize the potential complications.

Thromboembolism is one of the most common complications after TKA. Clinically, the application of the tourniquet is considered to be one of the most important risk factors for thromboembolism [40, 41]. Separated from the other complications, DVT was evaluated individually in this study, and there were no significant differences between the groups. Olivecrona et al. [39] demonstrated that using a tourniquet for more than 100 min in TKA would increase the incidence of DVT. The durations of tourniquet application were all less than 100 min in the included studies, and no difference was found in operative time between the groups. With increasing concerns about the negative effects related to DVT after TKA, perioperative DVT prophylaxis is now routinely applied. Thus, low incidence nature of this complication and the small sample size involved for comparison may partially account for the similar incidence of DVT in the two groups.

In the present meta-analysis, several important limitations should be recognized. First, the major limitation of this analysis is the small sample size of the included studies, and only seven RCTs were included, which may have caused imprecise outcomes. Second, some confounding factors such as the tourniquet pressure, method of thromboembolic prophylaxis, the type of anesthesia, analgesic methods, whether use drainage might influence the outcomes. Third, although some outcomes were reported in the included study, for the insufficient data and varied reporting of outcomes, some data were not sufficiently provided to perform meta-analysis, such as limb swelling. Finally, the follow-up period in most studies was relatively short, which might overlook the long-term outcomes, such as prosthetic fixation quality and joint function recovery.

## Conclusions

In conclusion, tourniquet application only during cementation increases intraoperative and total blood loss, while not increasing the number of transfusion and operation time. In addition, compared to long-duration tourniquet application, the limited use of a tourniquet results in less knee pain, less time needed to achieve straight-leg raise, and less minor complications following TKA, and functional recovery seems to be faster during the early rehabilitation period. Considering the relatively small sample size, no definitive conclusions can be drawn based on the current evidences. We hope our meta-analysis presented here will enable orthopedic surgeons to make an informed decision as to choose an appropriate tourniquet application strategy. Further large well-designed RCTs with extensive follow-up are still needed to validate this research.

## Abbreviations

BMI: Body mass index
CI: Confidence interval
DVT: Deep vein thrombosis
MD: Mean difference
OR: Odds ratios
POD 1: Post-operative day 1
RCTs: Randomized controlled trials
TKA: Total knee arthroplasty
VAS: Visual analogue score
